# Supplementary material for: Spatio-temporal dynamics of landscape use by the bumblebee Bombus pauloensis (Hymenoptera: Apidae) and its relationship with pollen provisioning
Source: PLoS One. 2020 Jul 8;15(7):e0216190. doi: 10.1371/journal.pone.0216190 (PMC7343142; doi:10.1371/journal.pone.0216190)
Supplement: S1 File — (DOCX) [file pone.0216190.s001.docx]

**S1-3 Video*. Bombus pauloensis* flight with 0.2 g radio transmitter attached in blueberry field agroecosystems in Entre Rios, Argentina.**

Detail of the foraging flight of queen of *B. pauloensis* with glued 0.2 g radio transmitter (ATS Series A2412) to the upper part of the abdomen.

Link 1: <https://doi.org/10.6084/m9.figshare.9757925.v1>

Link 2: <https://doi.org/10.6084/m9.figshare.9757928.v1>

Link 3: <https://doi.org/10.6084/m9.figshare.9757922.v1>
