# Supplementary figures and images for: Spatio-temporal dynamics of landscape use by the bumblebee Bombus pauloensis (Hymenoptera: Apidae) and its relationship with pollen provisioning
Source: PLoS One. 2020 Jul 8;15(7):e0216190. doi: 10.1371/journal.pone.0216190 (PMC7343142; doi:10.1371/journal.pone.0216190)

**S1 Figure. Technical specifications avian glue-on transmitter (model A2412).**


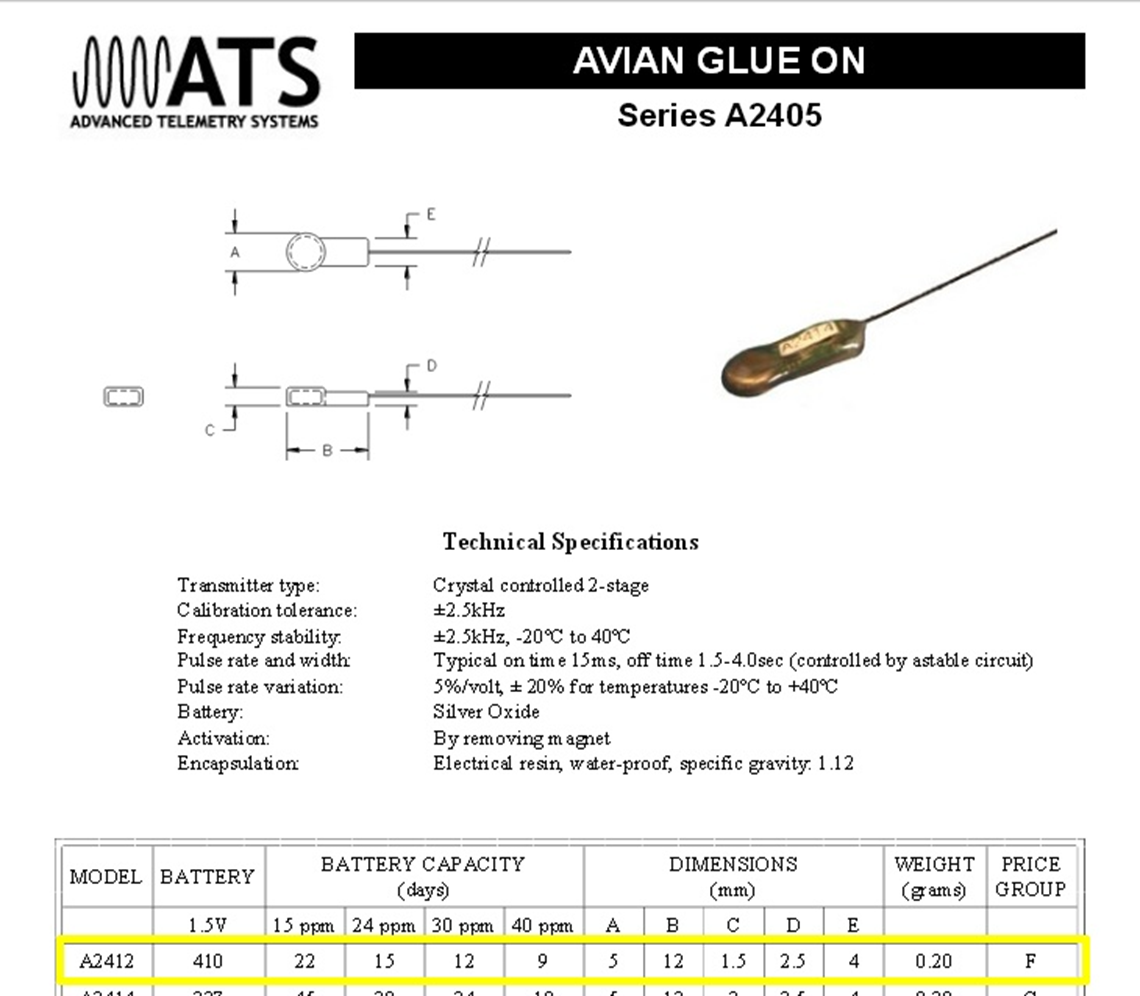

Supplement: S1 Fig — (DOCX) [file pone.0216190.s002.docx]
