# Supplementary material for: Spatio-temporal dynamics of landscape use by the bumblebee Bombus pauloensis (Hymenoptera: Apidae) and its relationship with pollen provisioning
Source: PLoS One. 2020 Jul 8;15(7):e0216190. doi: 10.1371/journal.pone.0216190 (PMC7343142; doi:10.1371/journal.pone.0216190)

**S2 Figure.** **Support information on the palynological characterization of pollen present in the *Bombus pauloensis* queens.**

Photos of flowers and associated pollen micrographs photographed on a standard optical microscope at 400x resolution. Flowering species are organized by taxonomic family.

**
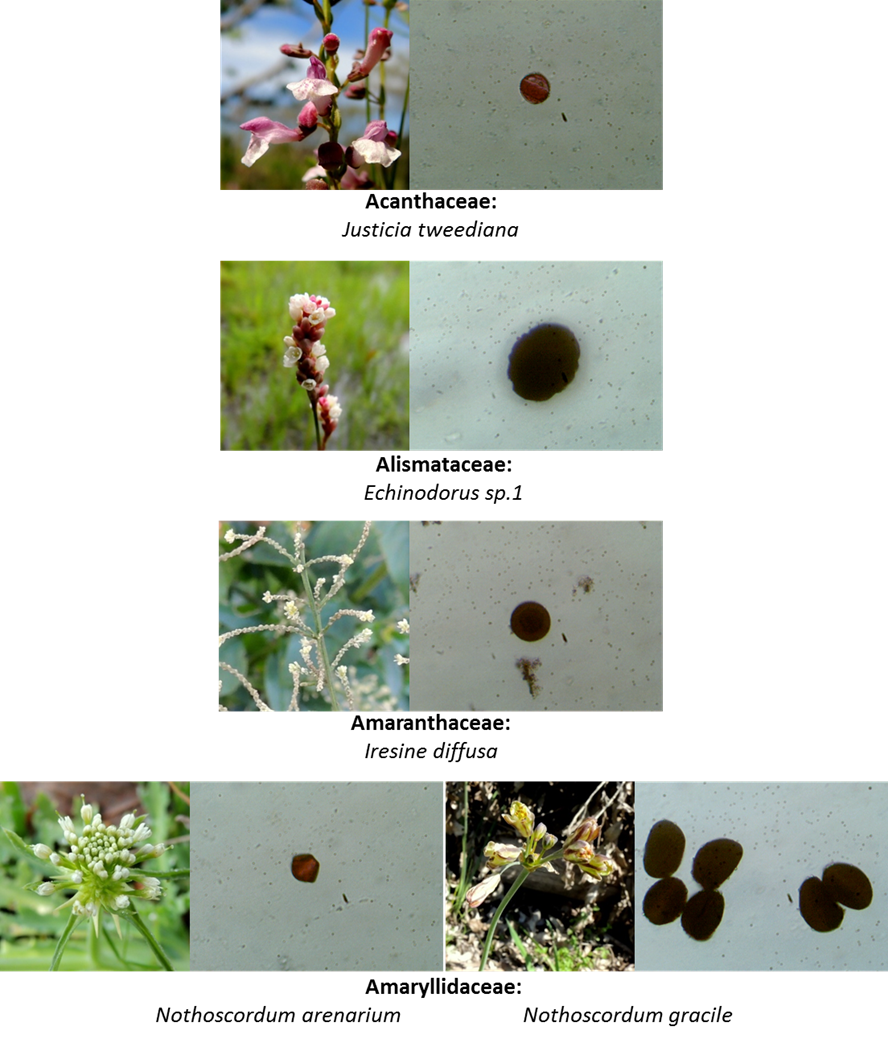
**

**
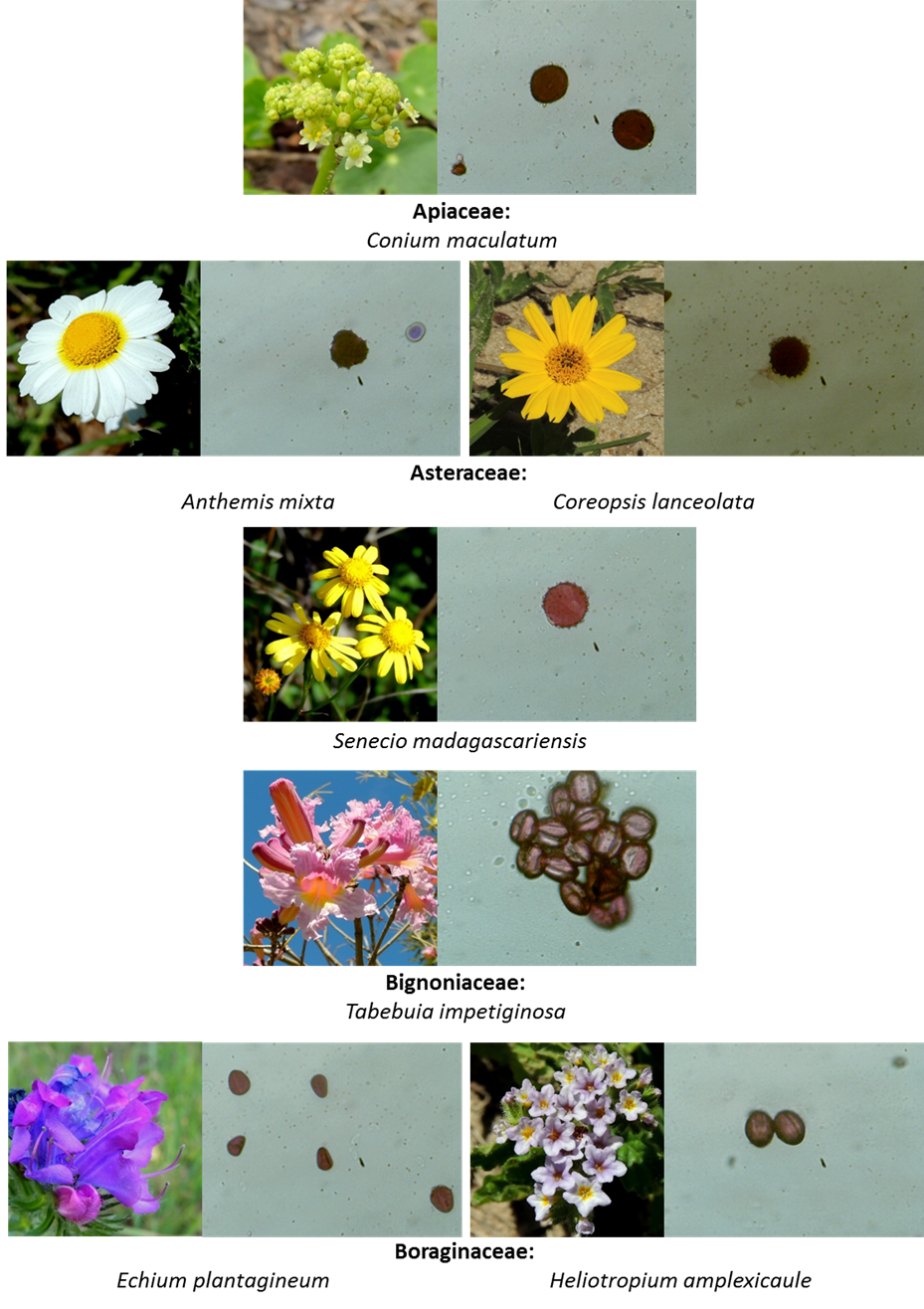
**

**
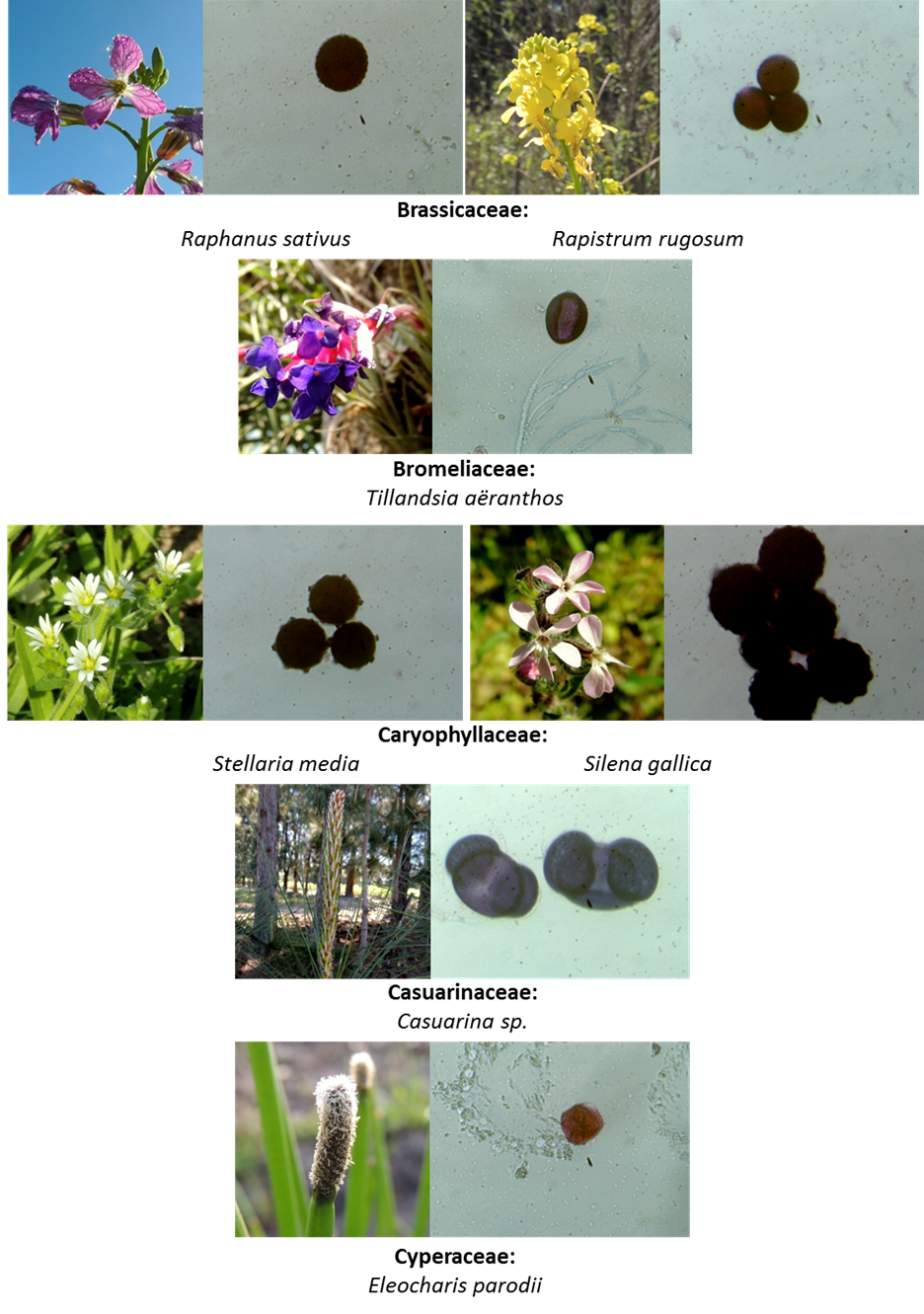
**

**
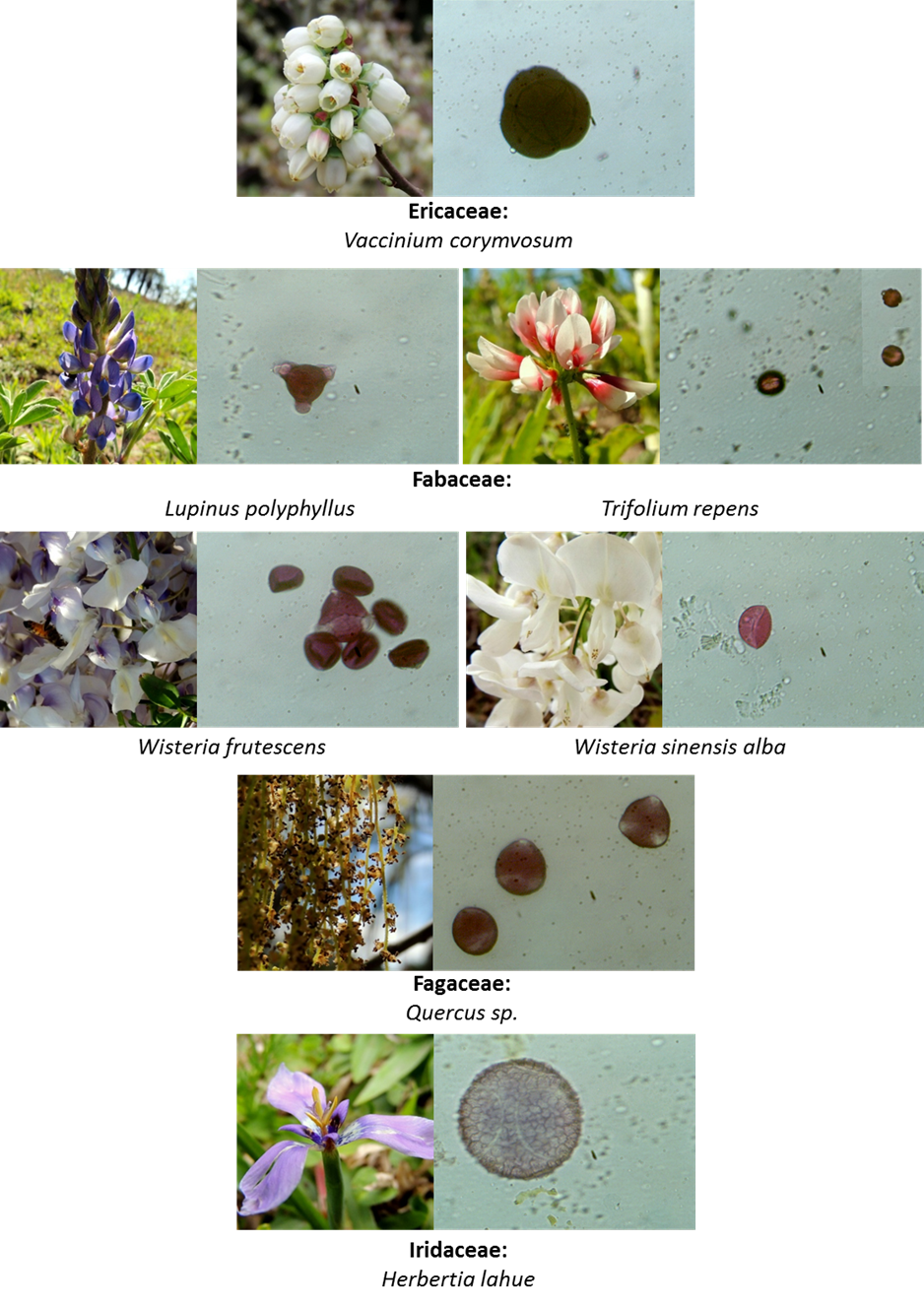
**

**
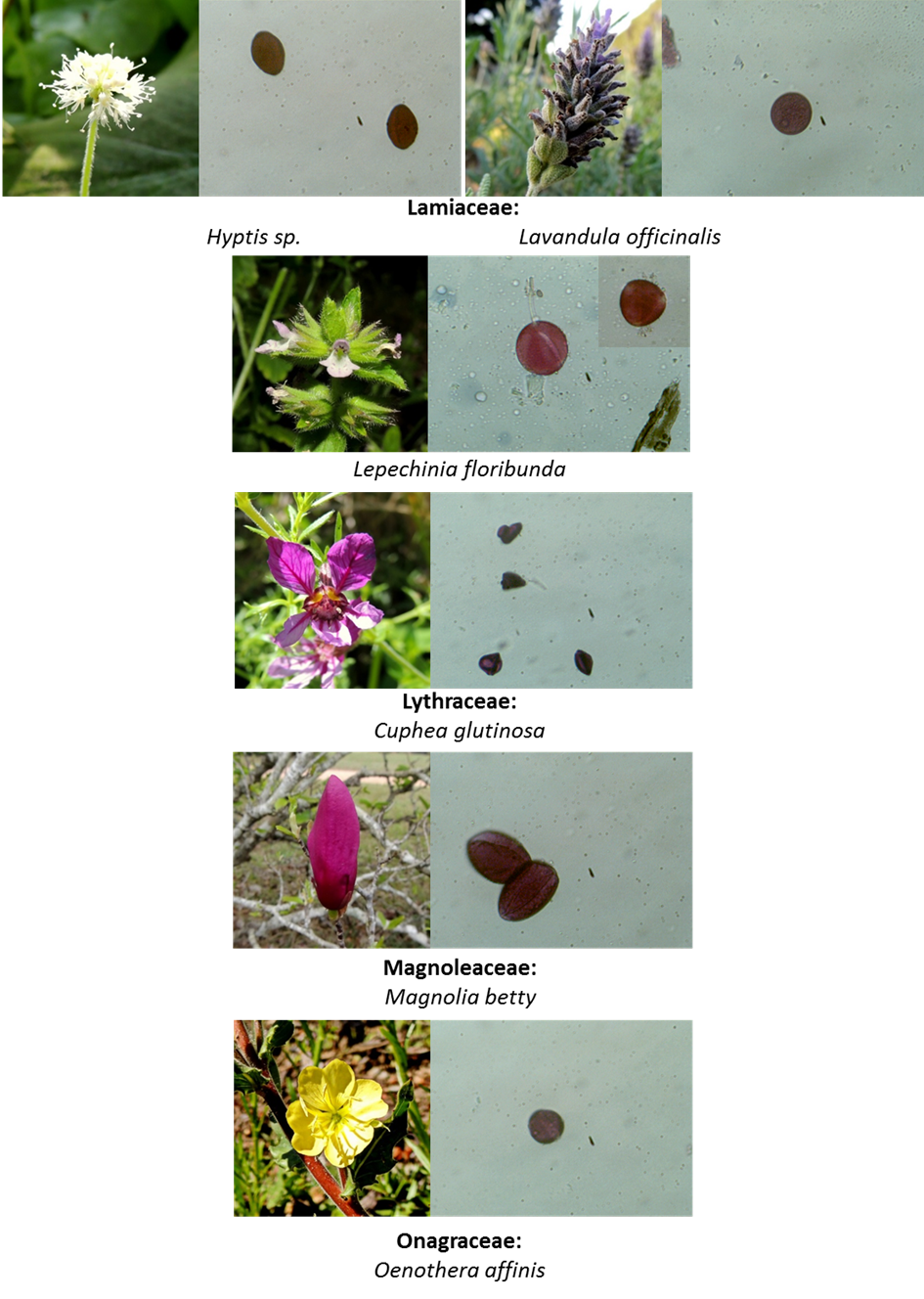
**


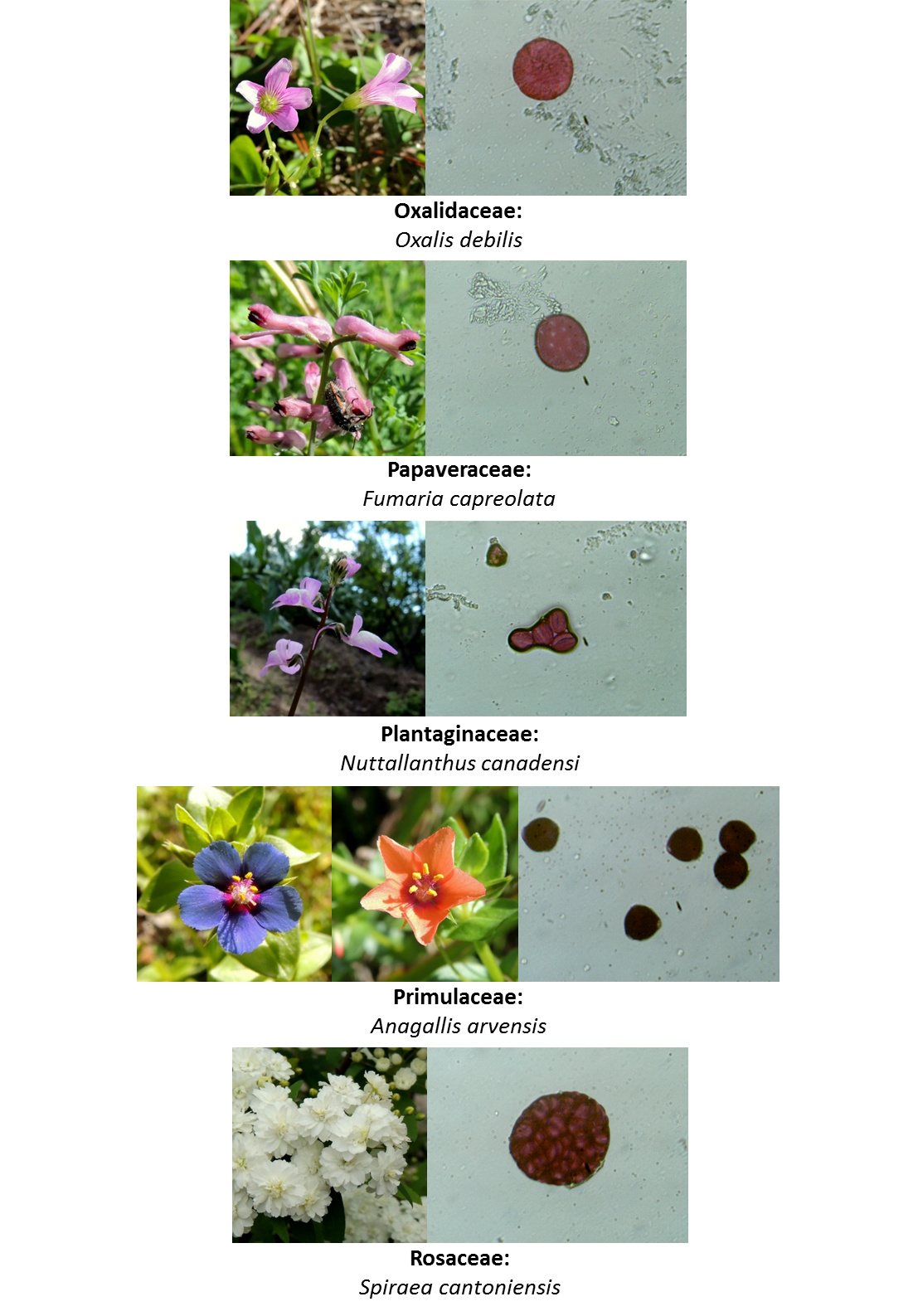


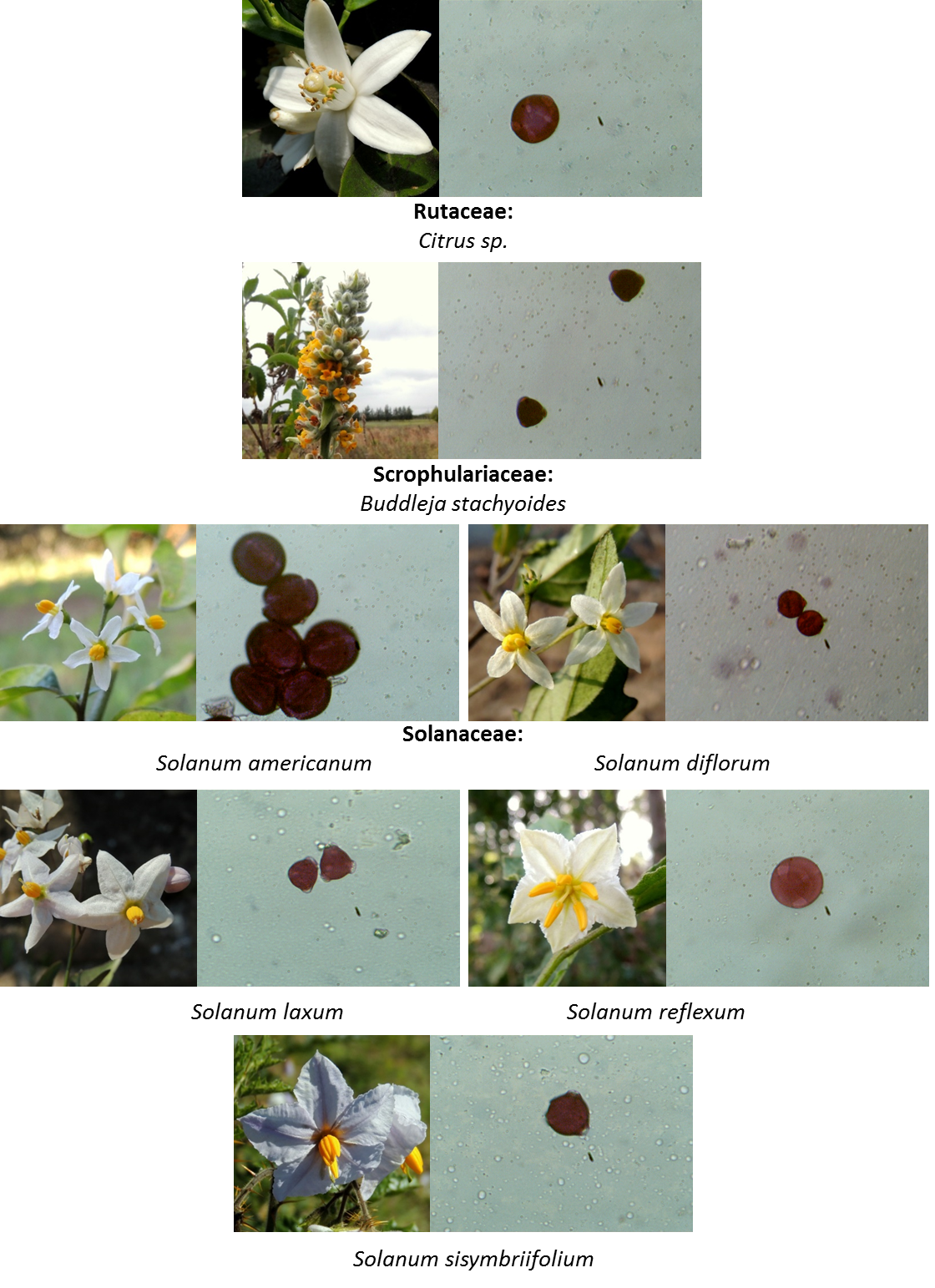


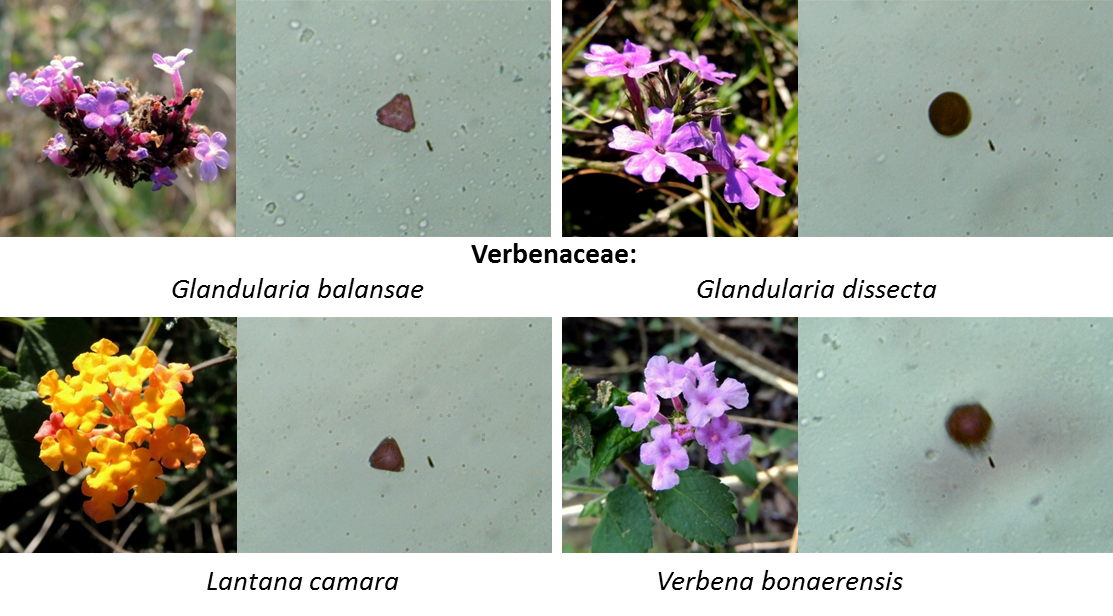

Supplement: S2 Fig — (DOCX) [file pone.0216190.s003.docx]
