# Supplementary material for: Spatio-temporal dynamics of landscape use by the bumblebee Bombus pauloensis (Hymenoptera: Apidae) and its relationship with pollen provisioning
Source: PLoS One. 2020 Jul 8;15(7):e0216190. doi: 10.1371/journal.pone.0216190 (PMC7343142; doi:10.1371/journal.pone.0216190)
Supplement: S1 Table — (DOCX) [file pone.0216190.s004.docx]

**S1 Table. Complementary information of the Kernel maps.**

| **S1 Table.** Description of the data set for the estimates kernel function for the before and after nesting stage. | | | | | | |
| --- | --- | --- | --- | --- | --- | --- |
|  | | | | | | |
| **Stage** | **Minimum** | **Maximum** | **Mean** | **Standard Deviation** | **Pixel Size** | **Original coating projection** |
| Before Nest | 0 | 7.1931 | 0.9835 | 1.2725 | 2.95771e-05/ -2.95771e-05 | -58.1311742342775091/ -31.3813778242775001 -58.1144631663745557/ -31.3665892686111683 |
| After Nest | 0 | 41.1550 | 2.4625 | 4.8623 | 2.63755e-05/ -2.63755e-05 | -58.1347181842774958/ -31.3734071842775002 -58.1186291365449321/ -31.3602194402344132 |
| Information obtained from the Metadata of QGIS 3.14.3 Essen, using a Quadratica (triponderated) Kernel function with a radius of 60 layer units. For the pre-nest stage, the kernel function was calculated from 152 waypoints and from the post-nest of 306 waypoints. | | | | | | |
