# Supplementary material for: Spatio-temporal dynamics of landscape use by the bumblebee Bombus pauloensis (Hymenoptera: Apidae) and its relationship with pollen provisioning
Source: PLoS One. 2020 Jul 8;15(7):e0216190. doi: 10.1371/journal.pone.0216190 (PMC7343142; doi:10.1371/journal.pone.0216190)
Supplement: S2 Table — (DOCX) [file pone.0216190.s005.docx]

**S2 Table. Complementary information of the Chi^2^-test between the observed locations and the random points.**

| **S2 Table.** Summary measures of the goodness-of-fit tests between the locations observed for each queen of *B. pauloensis* and the series of random points generated for each case. | | | |
| --- | --- | --- | --- |
|  | | | |
| **ID ^a^** | **Chi ^2^ Pearson** | **gl** | ***p*** |
| 105 | 29.1 | 3 | <0.0001 |
| 124 | 39.6 | 3 | <0.0001 |
| 144 | 48.15 | 3 | <0.0001 |
| 164 | 67.83 | 2 | <0.0001 |
| 185.3 | 26.18 | 4 | <0.0001 |
| 244 | 77.2 | 3 | <0.0001 |
| 244.2 | 17.2 | 3 | 0.0006 |
| 244.3 | 79.72 | 2 | <0.0001 |
| 264 | 78.42 | 2 | <0.0001 |
| 264.1 | 35.88 | 2 | <0.0001 |
| 304 | 0 | 2 | >0.9999 |
| 304.2 | 77.78 | 2 | <0.0001 |
| 364 | 69.79 | 3 | <0.0001 |
| 364.2 | 69.32 | 2 | <0.0001 |
| 385 | 19.46 | 2 | <0.0001 |
| 85 | 0.5 | 2 | 0.7797 |
| 5 | 14.88 | 3 | 0.0019 |
| ^a^ Identification code of each individual studied corresponding to the frequency of the transmitter fixed in his body. | | | |
