# Supplementary material for: Spatio-temporal dynamics of landscape use by the bumblebee Bombus pauloensis (Hymenoptera: Apidae) and its relationship with pollen provisioning
Source: PLoS One. 2020 Jul 8;15(7):e0216190. doi: 10.1371/journal.pone.0216190 (PMC7343142; doi:10.1371/journal.pone.0216190)
Supplement: S3 Table — (DOCX) [file pone.0216190.s006.docx]

**S3 Table. Complementary information of the proportional use in each LU.**

| **S3 Table.** Summary measures of the composition of LUs within each MCP. | | | | | | | | | |
| --- | --- | --- | --- | --- | --- | --- | --- | --- | --- |
|  | | | | | | | | | |
|  | | ***Proportion of each LU within MCP (%)*** | | | | ***Proportion of waypoint per LU*** | | | |
| **Stage** | **ID ^a^** | **BL** | **OF** | **FP** | **SN** | **BL** | **OF** | **FP** | **SN** |
| **Before nest** | 5 | 2.771 | 10.178 | 33.058 | 52.366 | 62.50 | 12.50 | 12.50 | 12.50 |
|  | 85 | 21.096 | 0.000 | 25.448 | 53.456 | 26.67 | 0.00 | 30.00 | 43.33 |
|  | 105 | 64.900 | 0.000 | 16.144 | 18.315 | 55.00 | 0.00 | 15.00 | 10.00 |
|  | 124 | 33.669 | 7.562 | 20.228 | 38.541 | 58.54 | 0.00 | 9.76 | 31.71 |
|  | 144 | 21.966 | 27.103 | 14.651 | 34.262 | 37.50 | 43.75 | 3.13 | 15.63 |
|  | 304 | 70.276 | 0.000 | 23.210 | 6.514 | 82.35 | 0.00 | 5.88 | 11.76 |
|  | 304.2 | 57.442 | 0.000 | 6.368 | 34.597 | 100.00 | 0.00 | 0.00 | 0.00 |
| **After nest** | 164 | 27.056 | 0.000 | 0.201 | 72.743 | 76.32 | 0.00 | 2.63 | 21.05 |
|  | 185.3 | 9.720 | 20.367 | 55.228 | 14.685 | 12.31 | 44.62 | 43.08 | 0.00 |
|  | 244 | 33.004 | 3.695 | 30.763 | 32.537 | 86.67 | 0.00 | 13.33 | 0.00 |
|  | 244.2 | 17.282 | 15.775 | 10.894 | 56.049 | 11.11 | 16.67 | 11.11 | 61.11 |
|  | 244.3 | 0.683 | 6.579 | 43.901 | 48.837 | 0.00 | 0.00 | 100.00 | 0.00 |
|  | 264 | 16.438 | 0.000 | 26.264 | 57.299 | 86.96 | 0.00 | 8.70 | 4.35 |
|  | 264.1 | 1.428 | 0.000 | 89.055 | 9.518 | 18.18 | 0.00 | 54.55 | 27.27 |
|  | 364 | 67.300 | 1.303 | 25.358 | 6.039 | 23.53 | 41.18 | 32.35 | 2.94 |
|  | 364.2 | 8.470 | 0.000 | 23.837 | 67.693 | 30.00 | 0.00 | 65.00 | 5.00 |
|  | 385 | 13.667 | 0.000 | 53.918 | 32.415 | 30.19 | 0.00 | 16.98 | 52.83 |
| ^a^ Identification code of each individual studied corresponding to the frequency of the transmitter fixed in his body. | | | | | | | | | |
| **References.** **BL**. Blueberry fields; **OF**. Area covered by other fruit trees (usually *Citrus sp.*); **FP**. Forest plantations and windbreaks; **SN**. Semi-natural areas. | | | | | | | | | |
