# Supplementary material for: Spatio-temporal dynamics of landscape use by the bumblebee Bombus pauloensis (Hymenoptera: Apidae) and its relationship with pollen provisioning
Source: PLoS One. 2020 Jul 8;15(7):e0216190. doi: 10.1371/journal.pone.0216190 (PMC7343142; doi:10.1371/journal.pone.0216190)
Supplement: S4 Table — (DOCX) [file pone.0216190.s007.docx]

**S4 Table. Complementary information of the proportional use of the floral resources by *Bombus pauloensis* queens (n=44).**

| **S4 Table.** Summary information of the relative palynological characterization of each individual bee analyzed. | | | | | | | | | | | | |
| --- | --- | --- | --- | --- | --- | --- | --- | --- | --- | --- | --- | --- |
|  | | | | | | | | | | | | |
|  | **Capture Date** | **N° Pollen spp.** | ***Vaccinium corymbosum*** | ***Justicia tweediana*** | ***Nothoscordum arenarium*** | ***Conium maculatum*** | ***Echium plantagineum*** | ***Cuphea glutinosa*** | ***Nuttalanthus canadensis*** | ***Buddleya stachyoides*** | ***Solanum sisymbriifolium*** | ***Others (45 spp) ^a^*** |
| **Early flower** | 20/07/2016 | 11 | 35 | 1 | 18 | 0 | 5 | 9 | 15 | 0 | 3 | 14 |
|  | 22/07/2016 | 11 | 35 | 8 | 8 | 0 | 4 | 36 | 0 | 0 | 0 | 9 |
|  | 22/07/2016 | 3 | 60 | 0 | 0 | 0 | 0 | 0 | 0 | 0 | 0 | 40 |
|  | 22/07/2016 | 9 | 25 | 1 | 0 | 0 | 0 | 1 | 2 | 1 | 1 | 69 |
|  | 22/07/2016 | 20 | 0 | 0 | 9 | 0 | 11 | 1 | 18 | 0 | 5 | 56 |
|  | 23/07/2016 | 5 | 84 | 0 | 0 | 0 | 0 | 0 | 0 | 0 | 0 | 16 |
|  | 28/07/2016 | 5 | 0 | 0 | 0 | 0 | 2 | 88 | 2 | 0 | 0 | 8 |
|  | 28/07/2016 | 13 | 70 | 1 | 0 | 0 | 4 | 0 | 6 | 0 | 0 | 19 |
|  | 29/07/2016 | 12 | 4 | 4 | 1 | 0 | 7 | 1 | 1 | 73 | 1 | 8 |
|  | 29/07/2016 | 11 | 27 | 24 | 13 | 2 | 12 | 0 | 0 | 5 | 0 | 17 |
|  | 29/07/2016 | 13 | 62 | 3 | 17 | 0 | 1 | 0 | 0 | 0 | 3 | 14 |
|  | 29/07/2016 | 10 | 77 | 6 | 5 | 0 | 1 | 0 | 5 | 0 | 0 | 6 |
|  | 29/07/2016 | 20 | 20 | 12 | 5 | 0 | 6 | 0 | 16 | 1 | 10 | 30 |
|  | 29/07/2016 | 16 | 58 | 4 | 9 | 0 | 1 | 1 | 5 | 0 | 0 | 22 |
|  | 29/07/2016 | 13 | 0 | 3 | 3 | 0 | 7 | 0 | 19 | 0 | 1 | 67 |
|  | 29/07/2016 | 14 | 22 | 3 | 5 | 0 | 3 | 4 | 15 | 0 | 4 | 44 |
|  | 02/08/2016 | 11 | 17 | 7 | 0 | 2 | 3 | 59 | 1 | 0 | 0 | 11 |
|  | 02/08/2016 | 2 | 84 | 0 | 0 | 0 | 0 | 0 | 0 | 0 | 0 | 16 |
|  | 04/08/2016 | 9 | 82 | 0 | 0 | 0 | 0 | 1 | 1 | 0 | 0 | 16 |
| **Peak flowering** | 09/08/2016 | 2 | 97 | 3 | 0 | 0 | 0 | 0 | 0 | 0 | 0 | 0 |
|  | 09/08/2016 | 2 | 98 | 0 | 0 | 0 | 0 | 0 | 0 | 0 | 0 | 2 |
|  | 11/08/2016 | 3 | 96 | 0 | 0 | 0 | 0 | 0 | 0 | 0 | 1 | 3 |
|  | 11/08/2016 | 17 | 4 | 16 | 13 | 1 | 7 | 2 | 30 | 1 | 4 | 22 |
|  | 16/08/2016 | 6 | 91 | 1 | 1 | 0 | 0 | 0 | 0 | 0 | 0 | 7 |
|  | 16/08/2016 | 3 | 99 | 1 | 0 | 0 | 0 | 0 | 0 | 0 | 0 | 0 |
|  | 16/08/2016 | 4 | 94 | 0 | 0 | 0 | 3 | 0 | 0 | 2 | 0 | 1 |
|  | 16/08/2016 | 18 | 44 | 3 | 2 | 0 | 4 | 0 | 4 | 0 | 5 | 38 |
|  | 18/08/2016 | 15 | 0 | 16 | 12 | 0 | 13 | 1 | 22 | 0 | 10 | 26 |
|  | 18/08/2016 | 3 | 95 | 0 | 0 | 0 | 0 | 0 | 0 | 0 | 4 | 1 |
|  | 18/08/2016 | 9 | 6 | 3 | 1 | 0 | 0 | 78 | 4 | 0 | 0 | 8 |
|  | 25/08/2016 | 20 | 17 | 6 | 3 | 0 | 9 | 10 | 18 | 0 | 0 | 37 |
| **Post-peak** | 13/09/2016 | 10 | 2 | 1 | 2 | 80 | 0 | 0 | 1 | 0 | 7 | 7 |
|  | 13/09/2016 | 9 | 1 | 3 | 4 | 67 | 0 | 0 | 2 | 0 | 2 | 21 |
|  | 13/09/2016 | 12 | 1 | 4 | 14 | 57 | 7 | 0 | 2 | 1 | 1 | 13 |
|  | 13/09/2016 | 10 | 1 | 0 | 6 | 0 | 0 | 3 | 1 | 5 | 0 | 84 |
|  | 13/09/2016 | 16 | 15 | 3 | 17 | 0 | 10 | 0 | 1 | 1 | 0 | 53 |
|  | 15/09/2016 | 12 | 0 | 0 | 4 | 13 | 0 | 0 | 3 | 40 | 1 | 39 |
|  | 15/09/2016 | 11 | 2 | 0 | 21 | 2 | 0 | 0 | 1 | 57 | 2 | 15 |
|  | 15/09/2016 | 8 | 0 | 0 | 3 | 2 | 0 | 1 | 0 | 57 | 0 | 37 |
|  | 15/09/2016 | 25 | 2 | 11 | 0 | 1 | 26 | 1 | 2 | 0 | 19 | 38 |
|  | 15/09/2016 | 7 | 2 | 13 | 14 | 0 | 23 | 0 | 2 | 0 | 0 | 46 |
|  | 15/09/2016 | 24 | 5 | 5 | 2 | 2 | 23 | 3 | 8 | 3 | 11 | 38 |
|  | 15/09/2016 | 22 | 2 | 5 | 14 | 0 | 26 | 10 | 3 | 1 | 12 | 27 |
|  | 15/09/2016 | 11 | 36 | 1 | 5 | 0 | 16 | 0 | 15 | 0 | 1 | 26 |
| The absolute values of the nine species of plants best represented in the body pollen of *B. pauloensis* queens are presented. | | | | | | | | | | | | |
| ^a^ The 45 species with less than 2% occurrence in total are grouped. | | | | | | | | | | | |  |
