# Supplementary material for: Spatio-temporal dynamics of landscape use by the bumblebee Bombus pauloensis (Hymenoptera: Apidae) and its relationship with pollen provisioning
Source: PLoS One. 2020 Jul 8;15(7):e0216190. doi: 10.1371/journal.pone.0216190 (PMC7343142; doi:10.1371/journal.pone.0216190)
Supplement: S5 Table — (DOCX) [file pone.0216190.s008.docx]

**S5 Table. Review of published works that estimate the homing distances for *Bombus* species.**

| **S5 Table.** Review of published literature that estimate the maximum average homing distances of species of the *Bombus* genus through different methodologies. | | |
| --- | --- | --- |
|  | | |
| **Authors** | ***Bombus* species** | **Mean max. homing distance (m)** |
| **Walther-Hellwig & Frankl (2000) [78] (Workers. queens and males)** | *B. muscorum* | 55 |
|  | *B. terrestris* | 663 |
|  | *B. lapidarius* | 260 |
| **Knight et al (2005) [85]** | *B. terrestris* | 758 |
|  | *B. pascuorum* | 449 |
|  | *B. lapidarius* | 450 |
|  | *B. pratorum* | 674 |
| **Wolf & Moritz (2008) [86]** | *B. terrestris* | 267.2 |
| **Hagen et al (2011) [56]** | *B. terrestris* | 2500 |
|  | *B. ruderatus* | 1900 |
|  | *B. hortorum (3 queens)* | 1300 |
| **Carvell et al (2012) [89]** | *B. lapidarius* | 755 |
|  | *B. pascuorum* | 775 |
| **Rao & Strange (2012) [87]** | *B. vosnesenskii* | 3500 |
| **Geib et al (2015) [71]** | *B. balteatus* | 85.4 |
|  | *B. flavifrons* | 23.8 |
|  | *B. bifarius* | 110.25 |
|  | *B. sylvicola* | 74.7 |
| **Redhead et al (2016) [88]** | *B. terrestris* | 551 |
|  | *B. lapidarius* | 536 |
|  | *B. ruderatus* | 501 |
|  | *B. hortorum* | 336 |
|  | *B. pascuorum* | 272 |
| In the event that it is not specified otherwise. the individuals used were workers.  [89] Carvell C, Jordan WC, Bourke AF, Pickles R, Redhead JW & Heard MS. Molecular and spatial analyses reveal links between colony‐specific foraging distance and landscape‐level resource availability in two bumblebee species. Oikos. 2012; 121(5), 734-742.  <https://doi.org/10.1111/j.1600-0706.2011.19832.x> | | |
